# Pararespiratory and paradigestive lymph node metastases in esophageal squamous cell carcinoma: predicting survival and refining the N staging system

**DOI:** 10.1186/s12885-023-11055-2

**Published:** 2023-07-24

**Authors:** Dong Tian, Kai-Yuan Jiang, Yu-Shang Yang, Hao-Ji Yan, Rui-Xuan Yu, Heng Huang, Shun-Hai Jian, Hai-Ning Zhou, Hong-Ying Wen, Long-Qi Chen

**Affiliations:** 1 Department of Thoracic Surgery, West China Hospital, Sichuan University, 37 Guoxue Alley, Chengdu, 610041 China; 2 Department of Cardiothoracic Intensive Care Unit, Affiliated Hospital of North Sichuan Medical College, Nanchong, 637000 China; 3 Department of Surgery, Tohoku University Graduate School of Medicine, Sendai, 980-8575 Japan; 4 College of Medical Imaging, North Sichuan Medical College, Nanchong, 637000 China; 5 Department of Thoracic Oncology, Cancer Center, West China Hospital, Sichuan University, Chengdu, 610041 China; 6 Department of Pathology, Affiliated Hospital of North Sichuan Medical College, Nanchong, 637000 China; 7 Department of Thoracic Surgery, Suining Central Hospital, Suining, 629000 China

**Keywords:** Esophageal squamous cell carcinoma, Lymph node metastasis, Pararespiratory, Paradigestive

## Abstract

**Background:**

The site of lymph node metastasis (LNM) may affect the prognosis of patients with esophageal squamous cell carcinoma (ESCC). To investigate the prognoses of pararespiratory and paradigestive LNM and to propose a novel N (nN) staging system that integrates both the LNM site and count.

**Methods:**

This study was a multicenter, large-sample, retrospective cohort study that included ESCC patients with LNM between January 2014 and December 2019 from three Chinese institutes. Patients were set into training (two institutes) and external validation (one institute) cohorts. The primary outcomes were survival differences in LNM site and the development of novel nodal staging system. The overall survival (OS) of patients with pararespiratory LNM only (Group A), paradigestive LNM only (Group B), and both sites (Group C) was evaluated by Kaplan-Meier. Cox proportional hazards models were used to identify the independent prognostic factors. An nN staging system considering both the LNM site and count was developed and evaluated by the area under the receiver operating characteristic curve (AUC).

**Results:**

In total, 1313 patients were included and split into training (n = 1033) and external validation (n = 280) cohorts. There were 342 (26.0%), 568 (43.3%) and 403 (30.7%) patients in groups A, B and C, respectively. The OS of patients with pararespiratory and patients with paradigestive LNM presented significant differences in the training and validation cohorts (P < 0.050). In the training cohort, LNM site was an independent prognostic factor (hazard ratio: 1.58, 95% confidence intervals: 1.41–1.77, P < 0.001). The nN staging definition: nN1 (1–2 positive pararespiratory/paradigestive LNs), nN2 (3–6 pararespiratory LNs or 1 pararespiratory with 1paradigestive LN), nN3 (3–6 LNs with ≥ 1 paradigestive LN), nN4 (≥ 7 LNs). Subsets of patients with different nN stages showed significant differences in OS (P < 0.050). The prognostic model of the nN staging system presented higher performance in the training and validation cohorts at 3-year OS (AUC, 0.725 and 0.751, respectively) and 5-year OS (AUC, 0.740 and 0.793, respectively) than the current N staging systems.

**Conclusions:**

Compared to pararespiratory LNM, the presence of paradigestive LNM is associated with worse OS. The nN staging system revealed superior prognostic ability than current N staging systems.

**Supplementary Information:**

The online version contains supplementary material available at 10.1186/s12885-023-11055-2.

## Introduction

Esophageal cancer ranks 6th in mortality among all cancers with 544, 000 new deaths occurring in 2020 [1]. The main prevalent histological type of esophageal cancer is esophageal squamous cell carcinoma (ESCC), constituting 90.0% of cases in China [2]. Lymph node metastasis (LNM) is one of the common metastatic routes of ESCC, exhibiting characteristics of continuous, bidirectional, and skip metastases, which significantly affects survival [3, 4]. Accurate node (N) staging is crucial for predicting patient prognosis and formulating optimal postoperative treatments [5].

The 8th edition American Joint Committee on Cancer (AJCC) & The Union for International Cancer Control (UICC) and the 11th edition of the Japan Esophageal Society (JES) tumor-node-metastasis (TNM) staging systems for esophageal cancer are widely used in current clinical work. However, the definitions of N staging are extremely different between the two staging systems. The AJCC/UICC TNM system defines the N staging based on the count of LNM [6]. In the JES TNM system, there are separate N staging criteria for tumors at different locations, and this staging is defined by the spread of LNM rather than the count [7]. To date, studies have reported modifications in N staging, mainly in terms of the metastatic LN ratio (mLNR, metastatic LNs/examined LNs), number of positive LN stations, and positive LN station ratio (positive LN stations/examined LN stations) [8–10]. Nevertheless, no study has investigated the association of LNM site with N stage.

A previous study demonstrated that the paraesophageal, paracardial, and left gastric artery LNs presented higher metastatic rates than supraclavicular and paratracheal LNs [11]. Furthermore, ESCC patients with left gastric artery and middle paraesophageal LNM had poor overall survival (OS), revealing the potential differences in survival based on LNM site [12, 13]. However, it is still unclear whether ESCC patients with LNM around the respiratory system and digestive system have favorable survival outcomes after curative esophagectomy. Hence, we first defined pararespiratory and paradigestive LN stations on the basis of the 8th edition AJCC/UICC system and aimed to investigate the prognoses of patients with LNM at the abovementioned sites. In addition, we attempted to develop a new N (nN) staging system that integrates both the site and count of LNM.

## Methods

### Study population

**Fig. 1:**
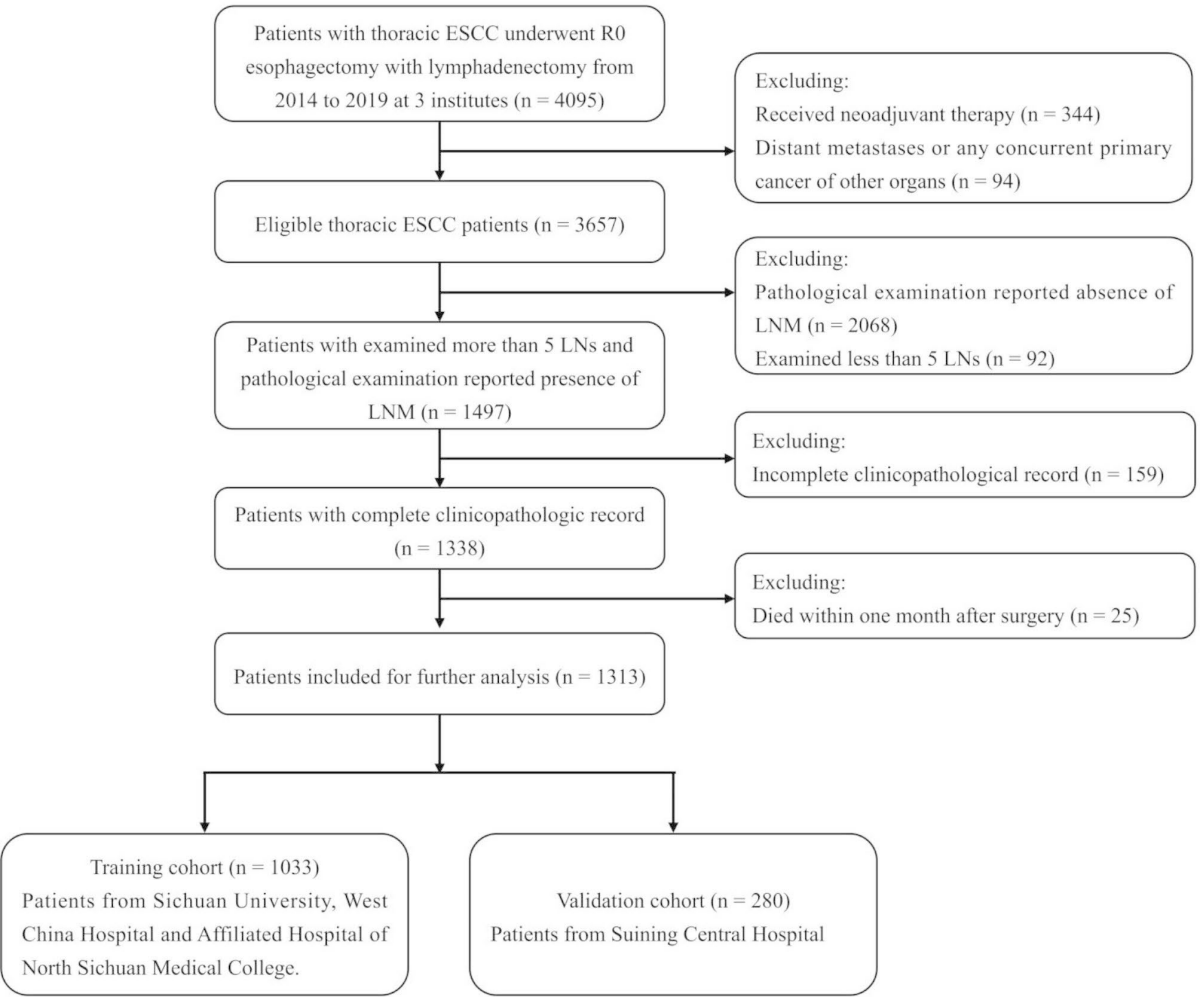
**The flow chart for patient inclusion and exclusion** ESCC, esophageal squamous cell carcinoma; LN, lymph node; LNM, lymph node metastasis

This study was a multicenter, retrospective study that included ESCC patients with LNM between January 2014 and December 2019 at three institutes. Patients were set in a training cohort (Sichuan University, West China Hospital and Affiliated Hospital of North Sichuan Medical College) and an external validation cohort (Suining Central Hospital). The inclusion criteria were as follows. (1) primary thoracic ESCC, (2) esophagectomy with LN dissection, (3) complete resection (R0) and (4) available pathological results for LNs. Patients with cervical ESCC were excluded from the study because of the limited cases, and the fact that upper abdominal lymph node dissection was not performed. In total, 4095 consecutive patients with thoracic ESCC were initially collected. We excluded 344 patient who received preoperative neoadjuvant therapy, 94 with distant metastasis or any concurrent primary cancer of other organs, 92 examined less than 5 LNs, 2068 with absence of LNM from pathology, 159 with incomplete clinicopathological records, and 25 with death in one month after surgery; thus, 1313 patients were ultimately enrolled for further analysis. The flow chart of selection is shown in Fig. 1. The study was performed in accordance with the Declaration of Helsinki. The Ethics Committees and Review Board of the Sichuan University, West China Hospital (No. 2019 − 632), Affiliated Hospital of North Sichuan Medical College (No. 2020ER181-1), and Suining Central Hospital (No. LLSNCH20200027) approved this study, and the need for patient informed consent was obtained.

### Surgical procedure

Patients with no contraindications underwent radical esophagectomy via left (Sweet) or right (McKeown or Ivor-Lewis) esophagectomy approaches depending on the tumor location with at least a two-field (thoracic and abdominal) lymphadenectomy. We did not perform routinely cervical LN dissection; only patients with suspicion of LNs involvement in the cervical field assessed by the preoperative computed tomography and ultrasound underwent three-field LN dissection. The LN stations were initially identified by surgeons during the operation according to the 8th AJCC/UICC TNM staging system.

### Definition

**Fig. 2:**
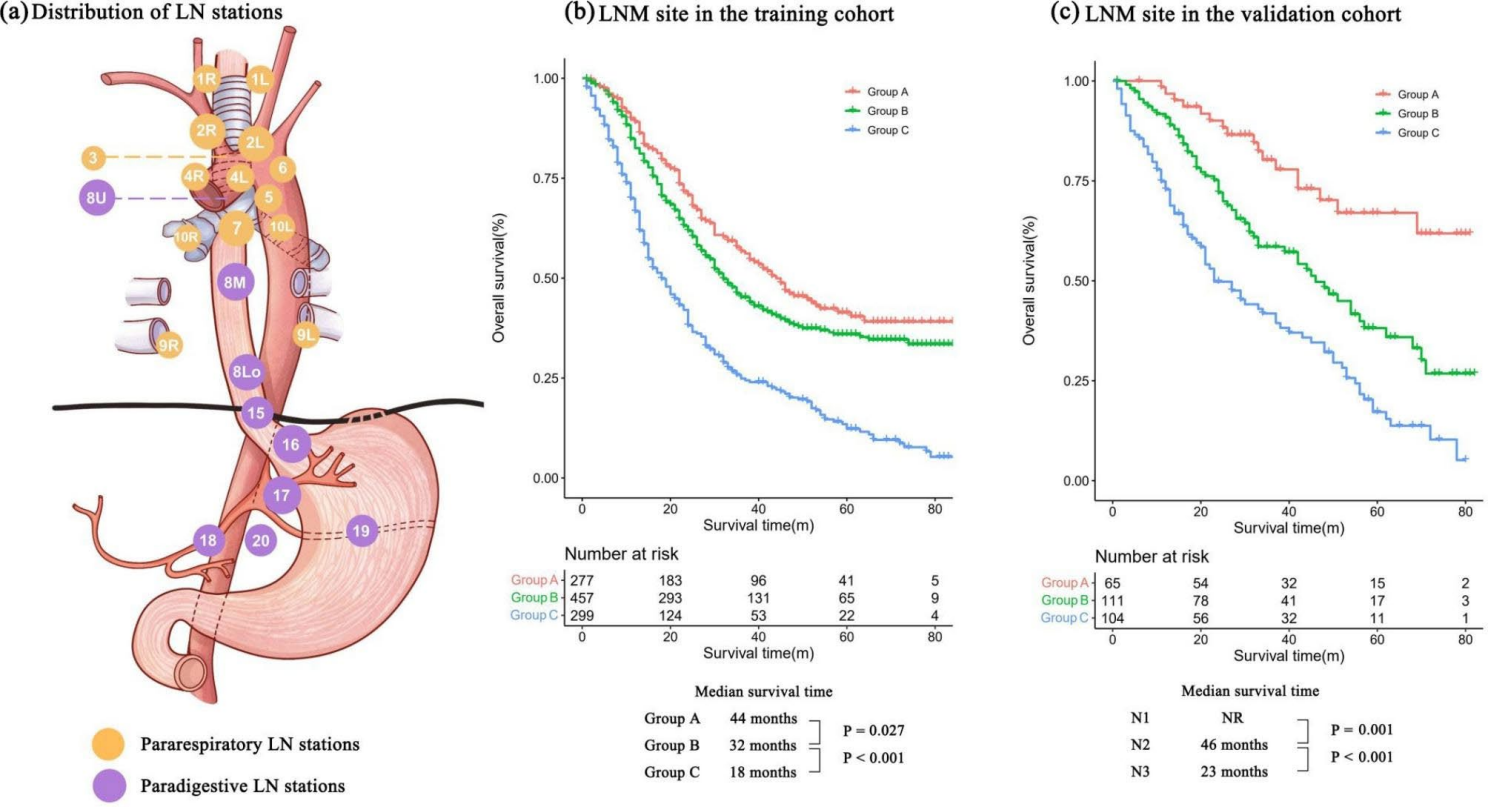
**Distribution of LN stations and survival curves for LNM site** **a**, Distribution of LN stations. **b**, Survival curve for LNM site in the training cohort (A vs. B: P = 0.027, A vs. C: P < 0.001, B vs. C: P < 0.001). **c**, Survival curve for LNM site in the validation cohort (A vs. B: P = 0.001, A vs. C: P < 0.001, B vs. C: P < 0.001). Group A, patients with pararespiratory LNM only; Group B, patients with paradigestive LNM only; Group C, patients with pararespiratory and paradigestive LNM both; LN, lymph node; LNM, lymph node metastasis; NR, not report

Pathological results were reevaluated (Y-SY and S-HJ) according to the 8th edition of the AJCC/UICC TNM staging system. Meanwhile, pathological tumor stage was assessed by JES TNM staging system based on available records. Stations 1, 2, 3, 4, 5, 6, 7, 9, and 10 are classified as pararespiratory LN stations, while stations 8, 15, 16, 17, 18, 19, and 20 are defined as paradigestive LN stations (Fig. 2a). The definition was based on their location relative to the respiratory and digestive tracts, as well as the naming convention in the AJCC/UICC TNM staging system. Patients with pararespiratory LNM only, paradigestive LNM only, and both sites were set as groups A, B and C, respectively. We tried to develop an nN staging system that integrated both the site and count of LNM on the basis of 8th AJCC/UICC N staging system. First, patients were divided into 9 groups by permutation and integration of LNM site (around the respiratory, digestive and both sites) and count (1–2, 3–6 and ≥ 7). Then, factorMerger method was performed to combine groups with similar distribution of survival curves into the same nN staging as previous study reported [14].

### Data collection and follow-up

Clinicopathological data included age, sex, preoperative comorbidity, postoperative complications, surgical approach, tumor location, tumor differentiation (G stage), T stage, AJCC/UICC N staging system, AJCC/UICC TNM staging system, JES N staging system, JES TNM staging system, count of examined LNs, and count of LNM. After surgery, follow-up was performed every 1–6 months, and the last follow-up time was conducted in July 2021. The overall follow-up duration was calculated from the date of surgery to the date of last follow-up or death. Patients lost to follow-up were censored at last contact.

### Statistical analysis

Statistical analyses were performed using IBM SPSS Statistics (version 25.0 Inc., Chicago, IL, USA) and the R programming language (version 4.0.2, Vienna, Austria). Clinicopathological data are described using descriptive statistics, frequency, and percentages for categorical variables. Continuous variables are expressed as the median (range). The Kaplan − Meier method with the log-rank test was used to estimate OS. For the training cohort, cox proportional hazards regression and binary logistic regression models were performed to determine the prognostic factors and risk factors for LNM site associated with worse survival, respectively. Only variables in the univariate analysis at P < 0.100 were included in the multivariate analysis. The odds ratio (OR), hazard ratio (HR) and 95% confidence interval (CI) were calculated. FactorMerger method with factor merge tree and survival plot was performed to determine the nN staging system in the training cohort. The area under the receiver operating characteristic curve (AUC) was used to investigate the predictive ability of different staging systems for 3- and 5-year OS in the training and validation cohorts. A P value < 0.050 was considered statistically significant.

## Results

### Clinicopathological characteristics

In total, 1313 patients (1060 male and 253 female) with a median age of 62 years (range: 37–81) were divided into a training cohort (n = 1033) and an external validation cohort (n = 280). There were 342 (26.0%), 568 (43.3%) and 403 (30.7%) patients in group A, group B and group C, respectively. The right surgical approach was performed in more than half of the cohorts (716, 54.5%). Most tumors originated from the middle portion of the esophagus (749, 57.0%), followed by the lower portion (440, 33.5%). Among all patients, 157 (12.0%), 602 (45.8%), and 554 (42.2%) cases were located at G1, G2 and G3, respectively. Tumor infiltration depth was predominantly at T2 and T3 in 341 (26.0%) and 601 (45.8%) patients, respectively. In our study, the majority of patients (991, 75.5%) had AJCC/UICC stage III disease. When the JES TNM staging system was applied, 928 (70.7%) patients had stage III disease. More detailed clinicopathologic information is shown in Table 1. Additionally, baseline characteristics for Group A, B and C in the training and validation cohort were showed in Supplementary Tables 1 and 2.

**Table 1:** Clinicopathological features of ESCC patients

Variables	No. of patients (%)/ Median (range)			P value
	All Cohort	Training Cohort	Validation Cohort	
Sex				**0.614**
Male	1060 (80.7)	831 (80.4)	229 (81.8)	
Female	253 (19.3)	202 (19.6)	51 (18.2)	
Age (years)	62 (37–81)	62 (37–81)	62 (40–79)	**0.954**
Preoperative comorbidity				**0.066**
Yes	794 (60.5)	638 (61.8)	156 (55.7)	
No	519 (39.5)	395 (38.2)	124 (44.3)	
Postoperative complications				**< 0.001**
Yes	355 (27.0)	256 (24.8)	99 (35.4)	
No	958 (73.0)	777 (75.2)	181 (64.6)	
Surgical approach				**0.560**
Left	597 (45.5)	474 (45.9)	123 (43.9)	
Right	716 (54.5)	559 (54.1)	157 (56.1)	
Location				**0.193**
Upper	124 (9.4)	92 (8.9)	32 (11.4)	
Middle	749 (57.0)	584 (56.5)	165 (58.9)	
Lower	440 (33.5)	357 (34.6)	83 (29.6)	
G stage				**< 0.001**
G1	157 (12.0)	114 (11.0)	43 (15.4)	
G2	602 (45.8)	445 (43.1)	157 (56.1)	
G3	554 (42.2)	474 (45.9)	80 (28.6)	
T stage				**< 0.001**
T1	196 (14.9)	126 (12.2)	70 (25.0)	
T2	341 (26.0)	220 (21.3)	121 (43.2)	
T3	601 (45.8)	527 (51.0)	74 (26.4)	
T4	175 (13.3)	160 (15.5)	15 (5.4)	
LNM site				**0.030**
Group A	342 (26.0)	277 (26.8)	65 (23.2)	
Group B	568 (43.3)	457 (44.2)	111 (39.6)	
Group C	403 (30.7)	299 (28.9)	104 (37.1)	
AJCC/UICC N staging system				**0.025**
N1	822 (62.6)	660 (63.9)	162 (57.9)	
N2	358 (27.3)	280 (27.1)	78 (27.9)	
N3	133 (10.1)	93 (9.0)	40 (14.3)	
AJCC/UICC TNM staging system				**0.001**
IIB	141 (10.7)	94 (9.1)	47 (16.8)	
III	991 (75.5)	799 (77.3)	192 (68.6)	
IVA	181 (13.8)	140 (13.6)	41 (14.6)	
JES N staging system				**0.290**
N1	538 (41.0)	413 (40.0)	125 (44.6)	
N2	600 (45.7)	478 (46.3)	122 (43.6)	
N3	127 (9.7)	100 (9.7)	27 (9.6)	
N4	48 (3.7)	42 (4.1)	6 (2.1)	
JES TNM staging system				**< 0.001**
II	337 (25.7)	220 (21.3)	117 (41.8)	
III	928 (70.7)	771 (74.6)	157 (56.1)	
IVa	48 (3.6)	42 (4.1)	6 (2.1)	
The count of examined LNs	17 (5–86)	18 (5–86)	12 (5–40)	**< 0.001**
The count of LNM	2 (1–47)	2 (1–47)	2 (1–16)	**0.104**

ESCC, esophageal squamous cell carcinoma; Group A, patients with pararespiratory LNM only; Group B, patients with paradigestive LNM only; Group C, patients with pararespiratory and paradigestive LNM both; AJCC/UICC, American Joint Committee on Cancer & The Union for International Cancer Control; JES, Japan Esophagus Society; LNM, lymph node metastasis; LN, lymph node

### LNM status

In all cohorts, the median number of involved and examined LNs were 2 (range: 1–47) and 17 (range: 5–86), respectively. The dominant stations of involved LNs were found at stations 8, 16 and 17, 2 and 7 with metastatic rates of 39.1%, 29.7%, 29.0%, 26.5% and 23.2%, respectively (Supplementary Fig. 1a). The total frequency of LNM observed around the respiratory and digestive systems was 931 vs. 1478. In addition, stations 1, 2, 8, 16 and 17 had higher mLNRs of 26.5%, 33.7%, 22.4%, 19.4% and 18.4%, respectively (Supplementary Fig. 1b). The total number of LNMs observed around the respiratory and digestive systems were 1455 and 2392, respectively.

### Overall survival

The median follow-up duration was 54 (range: 1–85) months. In the training cohort, the OS was significantly different among groups A, B and C, with median survival times of 40, 30 and 17 months, respectively (A vs. B: *P* = 0.014, A vs. C: *P* < 0.001, B vs. C: *P* < 0.001, Fig. 2b). In addition, a significant difference was also found in the validation cohort (A vs. B: *P* = 0.001, A vs. C: *P* < 0.001, B vs. C: *P* < 0.001, Fig. 2c). The subsets of patients in the training cohort based on AJCC/UICC staging (N1, N2 and N3) showed significant differences in OS (all P < 0.050, Supplementary Fig. 2a). When the JES N staging system was applied, patients with N1 disease were found to have better OS than those with N2, N3 and N4 disease (all P < 0.050), while no significant difference was observed among patients with N2, N3 and N4 disease (Supplementary Fig. 2b). Considering that most patients were in the TNM stage III (both the AJCC/UICC and JES staging systems), we further performed stratified analyses and found that patients with paradigestive LNM had poorer OS than those with pararespiratory LNM (all *P* < 0.050, Supplementary Fig. 3).

### Prognostic factors for overall survival in the training cohort

In the univariate analysis, the sex, preoperative comorbidities, postoperative complications, surgical approach, G stage, T stage, and LNM site were associated with OS (all P < 0.050). However, only male patients (HR 0.74, 95% CI 0.60–0.92, *P* = 0.007), postoperative complications (HR 1.34, 95% CI 1.11–1.61, *P* = 0.002), advanced T stage (HR 1.32, 95% CI 1.19–1.46, *P* < 0.001) and at least paradigestive LNM (HR 1.58, 95% CI 1.41–1.77, *P* < 0.001) were independent adverse prognostic factors in the multivariate analysis (Table 2).

**Table 2:** Univariate and multivariate analyses of overall survival in training cohort

Variables	Univariate		Multivariate	
	HR (95% CI)	P value	HR (95% CI)	P value
Sex (male/female)	0.73 (0.59–0.90)	0.003	0.74 (0.60–0.92)	0.007
Age (mean)	1.00 (0.99–1.01)	0.703		
Preoperative comorbidity (yes/no)	1.18 (1.01–1.40)	0.039	1.07 (0.91–1.27)	0.418
Postoperative complications (yes/no)	1.25 (1.00-1.49)	0.017	1.34 (1.11–1.61)	0.002
Surgical approach (left/right)	0.85 (0.73-1.00)	0.046	0.94 (0.80–1.10)	0.430
Location (upper/middle/lower)	1.05 (0.93–1.20)	0.427		
G stage (G1/G2/G3)	1.18 (1.05–1.33)	0.008	1.13 (0.99–1.27)	0.063
T stage (T1/T2/T3/T4)	1.42 (1.29–1.57)	< 0.001	1.32 (1.19–1.46)	< 0.001
LNM site (A/B/C)	1.64 (1.47–1.84)	< 0.001	1.58 (1.41–1.77)	< 0.001

HR, hazard ratio; CI, confidence interval; AJCC/UICC, American Joint Committee on Cancer & The Union for International Cancer Control; JES, Japan Esophagus Society; Group A, patients with pararespiratory LNM only; Group B, patients with paradigestive LNM only; Group C, patients with pararespiratory and paradigestive LNM both; LNM, lymph node metastasis

### Risk factors for paradigestive LNM in training cohort

Because paradigestive LNM resulted in worse prognoses, we further evaluated the risk factors for paradigestive LNM. In the univariate analysis, LNM around the digestive system showed significant associations with age, surgical approach, tumor location, and T stage (P < 0.050). After adjusting for significant clinical variables, the multivariable analysis revealed that younger age (OR 0.98, 95% CI 0.96-1.00, *P* = 0.020), left surgical approach (OR 0.50, 95% CI 0.36–0.70, *P* < 0.001) and tumor in the middle or lower esophagus (OR 2.84, 95% CI 2.16–3.74, *P* < 0.001) were independent risk factors for paradigestive LNM (Supplementary Table 3).

### The nN staging system

**Fig. 3:**
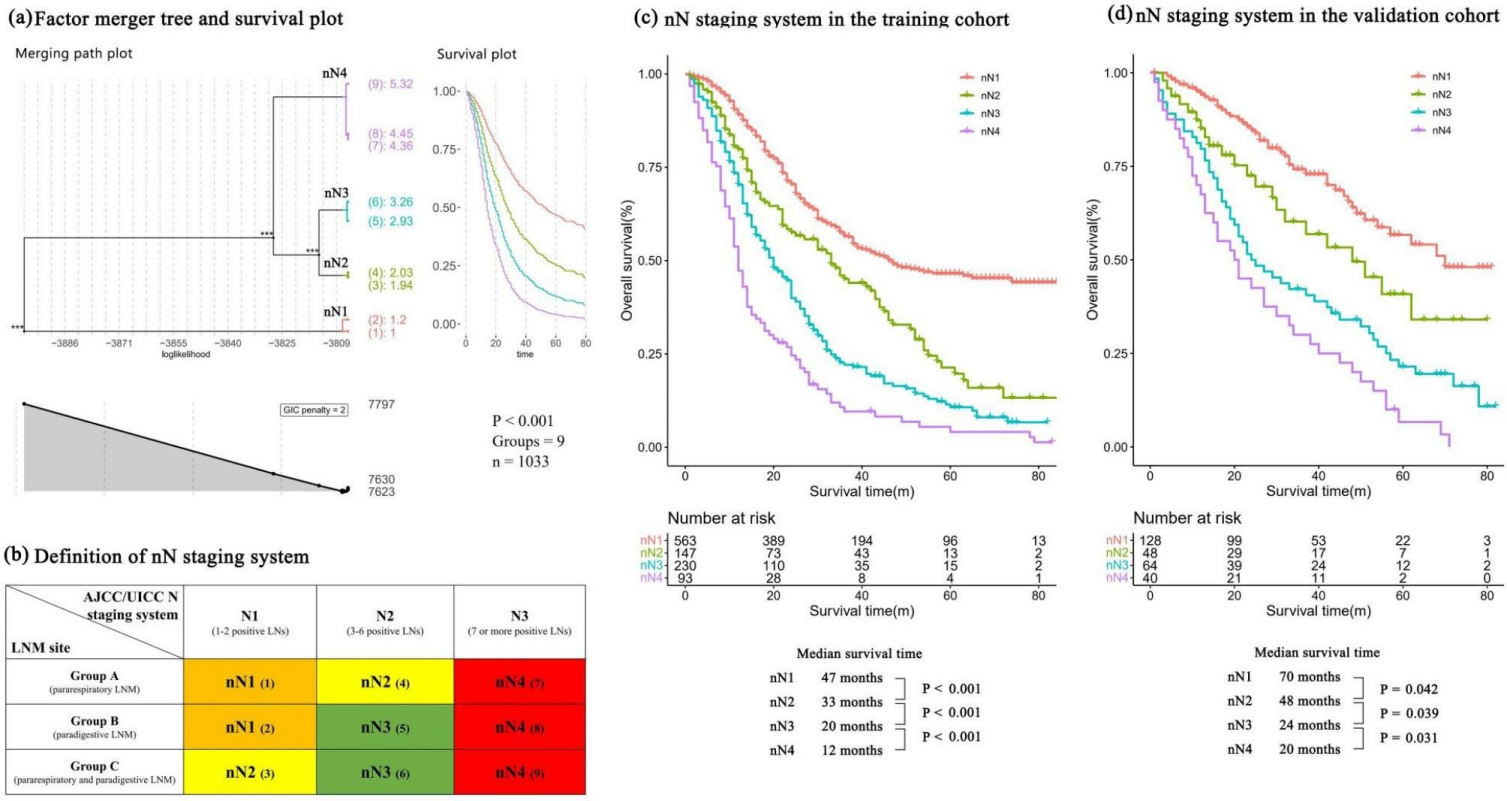
**Factor merger tree with survival plot, definition of nN staging system and Survival curves** **a**, Factor merger tree and survival plot. **b**, Definition of nN staging system. **c**, Survival curve for nN staging system in the training cohort (all P < 0.001). **d**, Survival curve for nN staging system in the validation cohort (nN1 vs. nN2: P = 0.042, nN1 vs. nN3: P < 0.001, nN1 vs. nN4: P < 0.001, nN2 vs. nN3: P = 0.039, nN2 vs. nN4: P < 0.001, nN3 vs. nN4: P = 0.031). AJCC/UICC, American Joint Committee on Cancer & The Union for International Cancer Control; LNM, lymph node metastasis; LN, lymph node

Considering the importance of the site and count of LNM, we integrated both criteria and developed a nN staging system based on merging path plot and survival plot in the training cohort (Fig. 3a). Briefly, the subsets of nN staging were defined as follows. nN1: 1–2 positive pararespiratory LNs or paradigestive LNs; nN2: 3–6 positive pararespiratory LNs or 1 positive pararespiratory LN with 1 paradigestive LN; nN3: at least one in 3–6 positive paradigestive LNs; and nN4: 7 or more positive LNs regardless of site (Fig. 3b). There were 563 (54.5%), 147 (14.2%), 230 (22.3%), and 93 (9.0%) patients with stage nN1, nN2, nN3, and nN4 disease, respectively. The nN staging system was presented as an independent prognostic factor in the multivariate Cox model analyses of OS (HR 1.59, 95% CI 1.48–1.71, *P* < 0.001, Supplementary Table 4). OS was significantly different among the above four groups, with the median survival times of 47, 33, 20 and 12 months, respectively (all *P* < 0.050, Fig. 3c). The validation cohort also showed a significant difference in OS between subsets of the nN staging system (all *P* < 0.050, Fig. 3d).

We further estimated the ability of the proposed nN staging system *versus* the current AJCC/UICC and JES N staging systems to predict survival. The AUCs of the 3- and 5-year Cox models are shown in Table 3. Briefly, the nN staging system demonstrated superior performance compared to the AJCC/UICC N staging and JES N staging systems at both 3-year OS (AUC, 0.725 vs. 0.716 and 0.648) and 5-year OS (AUC, 0.740 vs. 0.727 and 0.649) in the training cohort. Meanwhile, in external validation cohort, the nN staging system showed a better predictive accuracy than the other two N staging systems at 3-year OS (AUC, 0.751 vs. 0.741 and 0.741) and 5-year OS (AUC, 0.793 vs. 0.786 and 0.765).

**Table 3:** Area under the curve of the 3- and 5-year prognostic models based on different stagings

Staging systems	3-year		5-year	
	AUC	95% CI	AUC	95% CI
Training cohort				
AJCC/UICC N staging system	0.716	0.69–0.76	0.727	0.71–0.77
JES N staging system	0.648	0.63–0.69	0.649	0.64–0.71
nN staging system	0.725	0.69–0.76	0.740	0.71–0.77
Validation cohort				
AJCC/UICC N staging system	0.741	0.70–0.82	0.786	0.72–0.83
JES N staging system	0.741	0.67–0.80	0.765	0.66–0.78
nN staging system	0.751	0.72–0.83	0.793	0.74–0.85

AUC, area under the curve; CI, confidence interval; AJCC/UICC, American Joint Committee on Cancer & The Union for International Cancer Control; JES, Japan Esophagus Society

## Discussion

In this study, we first proposed pararespiratory and paradigestive LN stations, evaluated the survival of patients with LNM at the above two different sites, and developed a nN staging system for accurate OS prediction with external validation. The following findings were revealed. (a) Patients with paradigestive LNM had worse OS than those with pararespiratory LNM in both the training and validation cohorts. (b) LNM site was confirmed as an independent prognostic factor for OS. (c) We developed a nN staging system with four subsets that integrated both the site and count of LNM, and found that it predicted significantly different OS for patients. (d) Compared to the current AJCC/UICC and JES N staging systems, the nN staging system demonstrated higher performance in predicting 3- and 5-year OS, and was well validated in an external cohort.

Abundant longitudinal and transverse lymphatic networks constitute the lymphatic drainage system of the esophagus, providing an anatomical basis for LNM [15]. A systematic review and meta-analysis revealed that the most prevalent LNM sites in patients with ESCC were around paraesophageal and abdominal regions, possibly related to the primary site of the esophageal tumor [16]. In this study, the majority of involved LNs were located around the digestive system, which is consistent with the results of the meta-analysis.

Considering the potential differences in the clinicopathological significance of LNM at different sites, we further analyzed the OS of patients with LNM at different sites. Patients with paradigestive LNM had a worse survival than those with pararespiratory LNM in both the training and validation cohorts, which might indicate a higher malignancy rate in the former group. Previous studies have reported the prognoses of patients with LNM at various stations, but controversy remains. Liu et al. [13] and Ma et al. [17] reported that patients with subcarinal LNM had a worse OS than those with paraesophageal and left gastric artery LNM. In contrast, Hong and colleagues [12] reported that the 5-year survival rate of patients with paraesophageal LNM was significantly lower than that of patients with LNM around the recurrent laryngeal nerve and subcarinal nerve. The aforementioned controversies may result from the differences in research purpose, inclusion criteria and sample size. In addition to certain LN stations, previous studies have combined LN stations for further evaluation. Lin et al. [18] verified that the dominant LN stations (stations 1, 2, 8 and 16) of their cohort could better predict survival outcomes than nondominant LN stations in ESCC patients after surgery. The definition of dominant LN stations may vary across different institutes due to the patient’s inclusion criteria and lymphadenectomy region during surgery. Furthermore, they enrolled patients with cervical ESCC (28 cases in cervical and upper thoracic ESCC, accounting for 10.8%) which often presented more cervical LNM and led to an overestimation of station 1 metastasis, while only thoracic ESCC was included in our current study.

Recently, a large cohort by Harada and colleagues [19] assessed the prognostic influence of paratracheal LNM. They defined paratracheal LNs as stations 1, 2, and 4 according to the 8th AJCC/UICC staging system and found that paratracheal LNM was associated with shorter survival in resectable patients. However, they focused on esophageal adenocarcinoma and gastroesophageal junction adenocarcinoma, which may be pathophysiologically different from ESCC. In our study, we tried to extend the range of definitions as pararespiratory LNs and proposed the definition of paradigestive LNs which achieved satisfying results. Further analysis demonstrated that LNM site (paradigestive LNM/pararespiratory LNM) is an independent prognostic factor, and patients with paradigestive LNM had worse survival outcomes.

To our knowledge, previous studies have reported that age is not associated with LNM sites at stations 8 and 16 (8th AJCC/UICC staging system) [12, 13]. In this study, we revealed that younger patients were more likely to present paradigestive LNM. The reasons for this observation may include the higher tumor aggressiveness and baseline characteristics of younger patients in the current study. A majority of tumors in the younger patient (< 62 years, median) were located in the middle and lower thoracic esophagus (308/335), which consequently increases the risk of paradigestive LNM. Moreover, patients with ESCC originating from the middle and lower esophagus showed a higher rate of paradigestive LNM in the current cohort. This result is in accordance with Tachimori et al. [20] who found that more positive LNs were located in the perigastric and celiac regions, due to direct infiltration of tumors at the middle-lower esophagus. Interestingly, patients who underwent the left approach (sweet thoracotomy) in our study more frequently showed paradigestive LNM. This may be because of the limited examination of LNs located in the upper mediastinum when using the sweet thoracotomy [21, 22]. The pararespiratory LNM (23.4%) by the left approach may be underestimated, leading to a higher rate of paradigestive LNM (76.6%) (Supplementary Table 5).

Previous studies have reported that the N staging strategy in the 8th AJCC/UICC staging system sometimes fails to discriminate survival outcomes between N2 and N3 stage patients [8, 23, 24]. Therefore, there are ongoing efforts to refine the current N staging system to predict survival. However, the advantages are limited, and consensus has not been achieved. Ning et al. [8] and Peng et al. [11] reported that modified N categories based on the number of positive LN stations could better predict survival than the AJCC/UICC staging system. The rationale of using LN stations for a better prognostic prediction may help to limit imprecise LN calculations for multinodular fusion [25, 26]. Moreover, Fu and colleagues [9] revealed that the LN station ratio (number of positive LN stations/examined LN stations) category had superior predictive ability relative to the 8th AJCC/UICC N staging system. Recently, Zhang et al. [10] proposed the feasibility of modified N staging based on the ratio of positive to negative LNs. However, extended LN dissection would decrease the ratio and result in overestimation of survival. The JES N staging system seems more practically adaptable in describing the spread of esophageal cancer at different locations but failed to well predict survival in the current study and no revision based on JES N staging system has been proposed [27]. Moreover, Kunisaki et al. [28] reported that the number of LNMs was more significant for the survival outcomes than the sites.

In our study, both the LNM site and the AJCC/UICC N staging system (the count of LNM-based) were demonstrated to be ideal classifiers, although the two categorization principles were extremely different. We hypothesized that there might be more benefit from the integration of both classifiers and developed a nN staging system on the basis of the site and count of LNM. Our current proposal is extremely different from previous studies investigating the number of positive LN stations and mLNR [9, 11, 24]. Both our nN staging system and theirs presented a better or similar predictive performance compared to AJCC/UICC or JES N staging systems. However, our system is more comprehensive and convincing and has been validated in an external cohort.

The current study has several limitations that should be mentioned. First, this was a retrospective study with inevitable selection bias. Current findings should be further validated by a randomized controlled trial. Second, because the LN stations were identified during surgery according to the 8th AJCC/UICC TNM staging system, there may be errors in the reassessed JES N staging based on the available pathology reports. Third, some LN stations (stations 5, 6, 9) may be equivocal when defined as pararespiratory LNs in our study. Considering the small case number (15, 4, 45, respectively) and low LNM rate (1.1%, 0.3%, 3.4%, respectively), the results may not be affected by these stations. Lastly, future studies should consider including N0 patients to enhance the comprehensiveness and validation of the new N staging system.

## Conclusion

Our study is the first to report that the site of LNM is an independent prognostic factor and that patients with paradigestive LNM showed a worse OS than those with pararespiratory LNM. We developed a nN staging system according to the site and count of LNM, and the proposed nN staging system showed superior prognostic ability when compared to the 8th AJCC/UICC and 11th JES N staging systems, potentially providing an additional reference when formulating future guidelines for N staging.

## Electronic supplementary material

Below is the link to the electronic supplementary material.

Supplementary Material 1

## Data Availability

The datasets used or analysed during the current study available from the corresponding author on reasonable request.

## Abbreviations

AJCC: The American Joint Committee on Cancer
AUC: Area under the receiver operating characteristic curve
CI: Confidence interval
ESCC: Esophageal squamous cell carcinoma
HR: Hazard ratio
JES: The Japan Esophageal Society
LN: Lymph node
LNM: Lymph node metastasis
mLNR: Metastatic lymph node ratio
nN: Novel N
OR: Odds ratio
OS: Overall survival
TNM: Tumor-node-metastasis
UICC: The Union for International Cancer Control.
