# Supplementary material for: Pararespiratory and paradigestive lymph node metastases in esophageal squamous cell carcinoma: predicting survival and refining the N staging system
Source: BMC Cancer. 2023 Jul 24;23:695. doi: 10.1186/s12885-023-11055-2 (PMC10367276; doi:10.1186/s12885-023-11055-2)
Supplement: Supplementary file 1 — Supplementary Material 1 [file 12885_2023_11055_MOESM1_ESM.docx]

**Supplementary Material**

**Tables**

**Supplementary Table 1:** Clinicopathological features of ESCC patients for Groups A, B and C in training cohort

| Variables | No. of patients (%)/ Median (range) | | |
| --- | --- | --- | --- |
|  | Group A | Group B | Group C |
| Sex |  |  |  |
| Male | 217 (78.3) | 372 (81.4) | 242 (80.9) |
| Female | 60 (21.7) | 85 (18.6) | 57 (19.1) |
| Age (years) | 64 (37-80) | 61 (39-81) | 62 (38-79) |
| Preoperative comorbidity |  |  |  |
| Yes | 166 (59.9) | 276 (60.4) | 196 (65.6) |
| No | 111 (40.1) | 181 (39.6) | 103 (34.4) |
| Postoperative complications |  |  |  |
| Yes | 72 (26.0) | 124 (27.1) | 60 (20.1) |
| No | 205 (74.0) | 333 (72.9) | 239 (79.9) |
| Surgical approach |  |  |  |
| Left | 86 (31.0) | 230 (50.3) | 158 (52.8) |
| Right | 191 (69.0) | 227 (49.7) | 141 (47.2) |
| Location |  |  |  |
| Upper | 52 (18.8) | 21 (4.6) | 19 (6.4) |
| Middle | 172 (62.1) | 232 (50.8) | 180 (60.2) |
| Lower | 53 (19.1) | 204 (44.6) | 100 (33.4) |
| G stage |  |  |  |
| G1 | 31 (11.2) | 60 (13.1) | 23 (7.7) |
| G2 | 129 (46.6) | 209 (45.7) | 107 (35.8) |
| G3 | 117 (42.2) | 188 (41.1) | 169 (56.5) |
| T stage |  |  |  |
| T1 | 47 (17.0) | 58 (12.7) | 21 (7.0) |
| T2 | 71 (25.6) | 95 (20.8) | 54 (18.1) |
| T3 | 129 (46.6) | 244 (53.4) | 154 (51.5) |
| T4 | 30 (10.8) | 60 (13.1) | 70 (23.4) |
| AJCC/UICC N staging system |  |  |  |
| N1 | 221 (79.8) | 342 (74.8) | 97 (32.4) |
| N2 | 50 (18.1) | 99 (21.7) | 131 (43.8) |
| N3 | 6 (2.2) | 16 (3.5) | 71 (23.7) |
| AJCC/UICC TNM staging system |  |  |  |
| IIB | 39 (14.1) | 46 (10.1) | 9 (3.0) |
| III | 228 (82.3) | 380 (83.2) | 191 (63.9) |
| IVA | 10 (3.6) | 31 (6.8) | 99 (33.1) |
| JES N staging system |  |  |  |
| N1 | 113 (40.8) | 261 (57.1) | 39 (13.0) |
| N2 | 119 (43.0) | 166 (36.3) | 193 (64.5) |
| N3 | 31 (11.2) | 22 (4.8) | 47 (15.7) |
| N4 | 14 (5.1) | 8 (1.8) | 20 (6.7) |
| JES TNM staging system |  |  |  |
| II | 73 (26.4) | 119 (26.0) | 28 (9.4) |
| III | 190 (68.6) | 330 (72.2) | 251 (83.9) |
| IVa | 14 (5.1) | 8 (1.8) | 20 (6.7) |
| The count of examined LNs | 17 (5-86) | 18 (5-46) | 19 (5-73) |
| The count of LNM | 1 (1-15) | 1 (1-12) | 4 (2-47) |

ESCC, esophageal squamous cell carcinoma; Group A, patients with pararespiratory LNM only; Group B, patients with paradigestive LNM only; Group C, patients with pararespiratory and paradigestive LNM both; AJCC/UICC, American Joint Committee on Cancer & The Union for International Cancer Control; JES, Japan Esophagus Society; LNM, lymph node metastasis; LN, lymph node

**Supplementary Table 2:** Clinicopathological features of ESCC patients for Groups A, B and C in validation cohort

| Variables | No. of patients (%)/ Median (range) | | |
| --- | --- | --- | --- |
|  | Group A | Group B | Group C |
| Sex |  |  |  |
| Male | 53 (81.5) | 90 (81.1) | 86 (82.7) |
| Female | 12 (18.5) | 21 (18.9) | 18 (17.3) |
| Age (years) | 63 (48-75) | 61 (40-79) | 62 (50-79) |
| Preoperative comorbidity |  |  |  |
| Yes | 40 (61.5) | 58 (52.3) | 58 (55.8) |
| No | 25 (38.5) | 53 (47.7) | 46 (44.2) |
| Postoperative complications |  |  |  |
| Yes | 15 (23.1) | 41 (36.9) | 43 (41.3) |
| No | 50 (76.9) | 70 (63.1) | 61 (58.7) |
| Surgical approach |  |  |  |
| Left | 12 (18.5) | 74 (66.7) | 37 (35.6) |
| Right | 53 (81.5) | 37 (33.3) | 67 (64.4) |
| Location |  |  |  |
| Upper | 15 (23.1) | 3 (2.7) | 14 (13.5) |
| Middle | 42 (64.6) | 60 (54.1) | 63 (60.6) |
| Lower | 8 (12.3) | 48 (43.2) | 27 (26.0) |
| G stage |  |  |  |
| G1 | 10 (15.4) | 26 (23.4) | 7 (6.7) |
| G2 | 41 (63.1) | 57 (51.4) | 59 (56.7) |
| G3 | 14 (21.5) | 28 (25.2) | 38 (36.5) |
| T stage |  |  |  |
| T1 | 26 (40.0) | 27 (24.3) | 17 (16.3) |
| T2 | 22 (33.8) | 56 (50.5) | 43 (41.3) |
| T3 | 16 (24.6) | 25 (22.5) | 33 (31.7) |
| T4 | 1 (1.5) | 3 (2.7) | 11 (10.6) |
| AJCC/UICC N staging system |  |  |  |
| N1 | 49 (75.4) | 79 (71.2) | 34 (32.7) |
| N2 | 14 (21.5) | 25 (22.5) | 39 (37.5) |
| N3 | 2 (3.1) | 7 (6.3) | 31 (29.8) |
| AJCC/UICC TNM staging system |  |  |  |
| IIB | 20 (30.8) | 20 (18.0) | 7 (6.7) |
| III | 43 (66.2) | 84 (75.7) | 65 (62.5) |
| IVA | 2 (3.1) | 7 (6.3) | 32 (30.8) |
| JES N staging system |  |  |  |
| N1 | 32 (49.2) | 69 (62.2) | 24 (23.1) |
| N2 | 31 (47.7) | 33 (29.7) | 58 (55.8) |
| N3 | 2 (3.1) | 7 (6.3) | 18 (17.3) |
| N4 | 0 (0) | 2 (1.8) | 4 (3.8) |
| JES TNM staging system |  |  |  |
| II | 34 (52.3) | 61 (55.0) | 22 (21.2) |
| III | 31 (47.7) | 48 (43.2) | 78 (75.0) |
| IVa | 0 (0) | 2 (1.8) | 4 (3.8) |
| The count of examined LNs | 12 (5-40) | 12 (5-34) | 13 (5-33) |
| The count of LNM | 1 (1-7) | 1 (1-10) | 3 (2-16) |

ESCC, esophageal squamous cell carcinoma; Group A, patients with pararespiratory LNM only; Group B, patients with paradigestive LNM only; Group C, patients with pararespiratory and paradigestive LNM both; AJCC/UICC, American Joint Committee on Cancer & The Union for International Cancer Control; JES, Japan Esophagus Society; LNM, lymph node metastasis; LN, lymph node

**Supplementary Table 3:** Univariate and multivariate logistic analysis of risk factors for paradigestive LNM in the training cohort.

| Variables | Univariate | | Multivariate | |
| --- | --- | --- | --- | --- |
|  | OR (95% CI) | P value | OR (95% CI) | P value |
| Sex (male/female) | 0.83 (0.57-1.20) | 0.313 |  |  |
| Age (mean) | 0.97 (0.95-0.99) | 0.001 | 0.98 (0.96-1.00) | 0.020 |
| Preoperative comorbidity (yes/no) | 1.02 (0.75-1.38) | 0.900 |  |  |
| Surgical approach (left/right) | 0.44 (0.33-0.61) | <0.001 | 0.50 (0.36-0.70) | <0.001 |
| Location (upper/middle/lower) | 3.01 (2.30-3.95) | <0.001 | 2.84 (2.16-3.74) | <0.001 |
| G stage (G1/G2/G3) | 0.94 (0.75-1.17) | 0.554 |  |  |
| T stage (T1/T2/T3/T4) | 1.23 (1.03-1.45) | 0.019 | 1.08 (0.90-1.30) | 0.402 |

OR, odds ratio; CI, confidence interval; AJCC/UICC, American Joint Committee on Cancer & The Union for International Cancer Control; LNM, lymph node metastasis.

**Supplementary Table 4:** Univariate and multivariate analyses of overall survival in training cohort (Based on nN staging system)

| Variables | Univariate | | Multivariate | |
| --- | --- | --- | --- | --- |
|  | HR (95% CI) | P value | HR (95% CI) | P value |
| Sex (male/female) | 0.73 (0.59-0.90) | 0.003 | 0.81 (0.65-1.01) | 0.059 |
| Age (mean) | 1.00 (0.99-1.01) | 0.703 |  |  |
| Preoperative comorbidity (yes/no) | 1.18 (1.01-1.40) | 0.039 | 1.07 (0.90-1.26) | 0.451 |
| Postoperative complications (yes/no) | 1.25 (1.00-1.49) | 0.017 | 1.39 (1.16-1.67) | <0.001 |
| Surgical approach (left/right) | 0.85 (0.73-1.00) | 0.046 | 0.93 (0.79-1.09) | 0.349 |
| Location (upper/middle/lower) | 1.05 (0.93-1.20) | 0.427 |  |  |
| G stage (G1/G2/G3) | 1.18 (1.05-1.33) | 0.008 | 1.06 (0.94-1.20) | 0.360 |
| T stage (T1/T2/T3/T4) | 1.42 (1.29-1.57) | <0.001 | 1.26 (1.14-1.39) | <0.001 |
| nN staging system | 1.64 (1.47-1.84) | <0.001 | 1.59 (1.48-1.71) | <0.001 |

HR, hazard ratio; CI, confidence interval; AJCC/UICC, American Joint Committee on Cancer & The Union for International Cancer Control; JES, Japan Esophagus Society; Group A, patients with pararespiratory LNM only; Group B, patients with paradigestive LNM only; Group C, patients with pararespiratory and paradigestive LNM both; LNM, lymph node metastasis.

**Supplementary Table 5:** Distribution of LNM site in left and right surgical approach.

| Surgical approach | Pararespiratory LNM only  Cases (%) | Paradigestive LNM only  Cases (%) |
| --- | --- | --- |
| Left | 86 (27.2%) | 230 (72.8%) |
| Right | 191 (45.7%) | 227 (54.3%) |

LNM, lymph node metastasis.

**Figures**


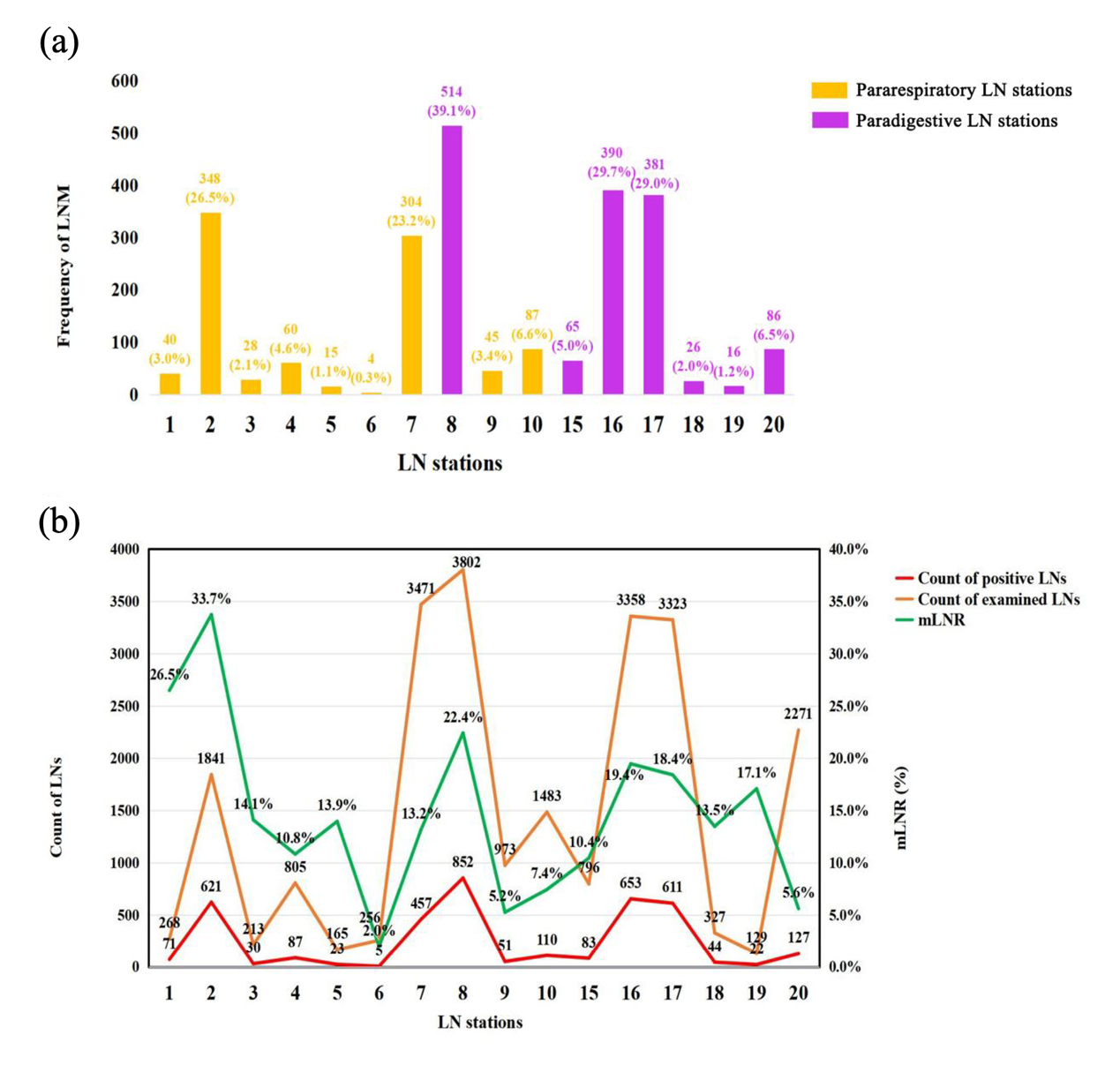


**Supplemental Figure 1:** Histogram of metastatic LN stations and Line chart of LNM count

Frequency of LNM = cases of positive LN station / total cases (1313); mLNR = count of positive LNs / count of examined LNs; LNM, lymph node metastasis; LN, lymph node; mLNR, metastatic lymph node ratio.


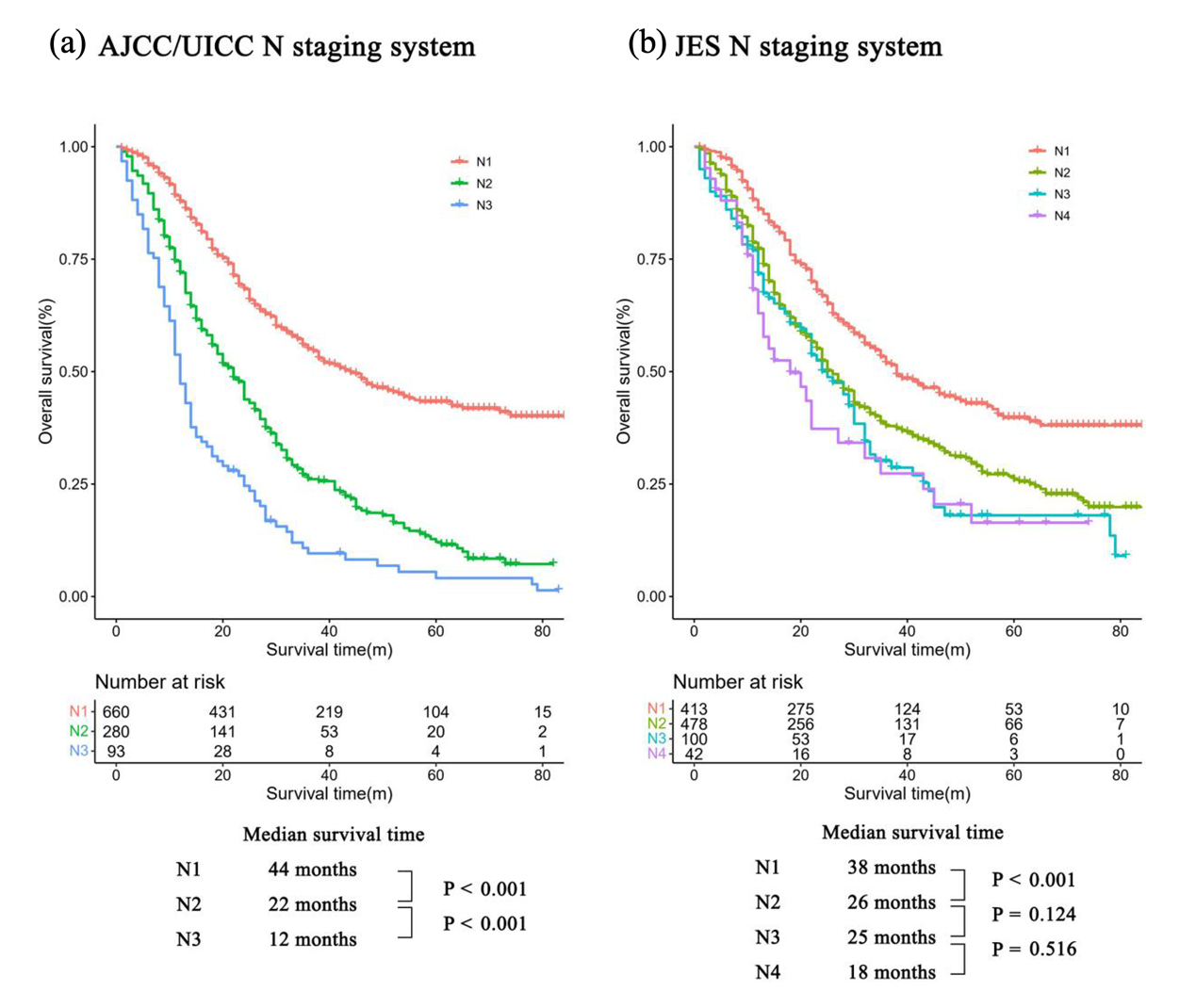


**Supplemental Figure 2:** The survival curves for AJCC/UICC and JES N staging systems in the training cohort.

a, AJCC/UICC N staging system (N1 vs N2: P < 0.001, N1 vs N3: P < 0.001, N2 vs N3: P < 0.001). b, JES N staging system (N1 vs N2: P < 0.001, N1 vs N3: P < 0.001, N1 vs N4: P < 0.001, N2 vs N3: P = 0.124, N2 vs N4: P = 0.079, N3 vs N4: P = 0.516). AJCC/UICC, American Joint Committee on Cancer & The Union for International Cancer Control; JES, Japan Esophagus Society.

**
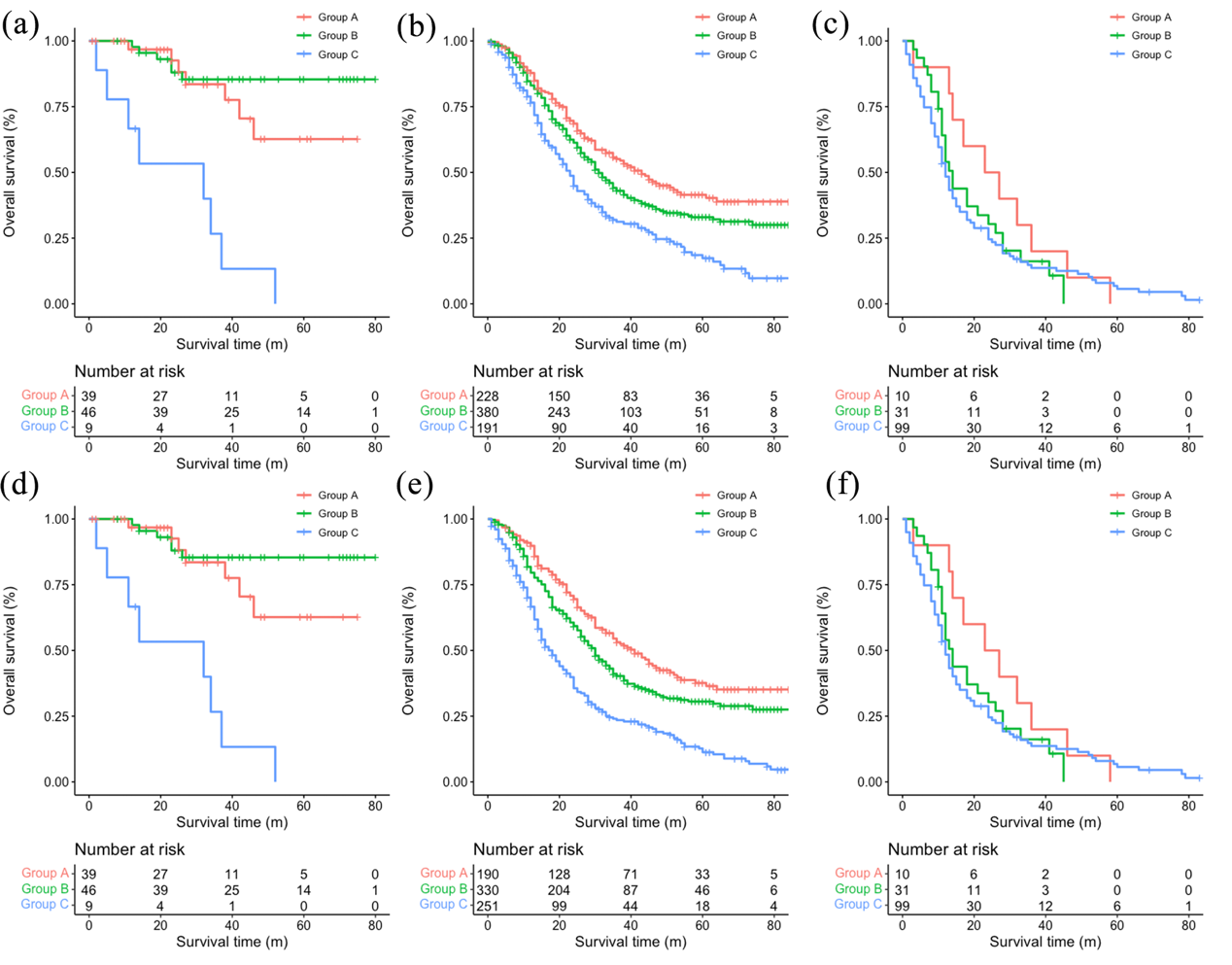
**

**Supplemental Figure 3:** Stratified analysis of LNM site in AJCC/UICC and JES TNM stage in the training cohort.

a, LNM site in AJCC/UICC TNM stage IIB (A vs B: P=0.227, A vs C: P<0.001, B vs C: P<0.001). b, LNM site in AJCC/UICC TNM stage III (A vs B: P=0.027, A vs C: P<0.001, B vs C: P<0.001). c, LNM site in AJCC/UICC TNM stage IVA (A vs B: P=0.183, A vs C: P=0.324, B vs C: P=0.632). d, LNM site in JES TNM stage II (A vs B: P=0.634, A vs C: P=0.011, B vs C: P=0.015). e, LNM site in JES TNM stage III (A vs B: P=0.017, A vs C: P<0.001, B vs C: P<0.001). f, LNM site in JES TNM stage IVA (A vs B: P=0.547, A vs C: P=0.087, B vs C: P=0.349). Group A, patients with pararespiratory LNM only; Group B, patients with paradigestive LNM only; Group C, patients with pararespiratory and paradigestive LNM both; AJCC/UICC, American Joint Committee on Cancer & The Union for International Cancer Control; JES, Japan Esophagus Society.
